# Effect of Subclinical Hypothyroidism on the Association between Hemoglobin A1c and Reduced Renal Function: A Prospective Study

**DOI:** 10.3390/diagnostics12020462

**Published:** 2022-02-11

**Authors:** Yuji Shimizu, Shin-Ya Kawashiri, Yuko Noguchi, Seiko Nakamichi, Yasuhiro Nagata, Takahiro Maeda, Naomi Hayashida

**Affiliations:** 1 Department of General Medicine, Nagasaki University Graduate School of Biomedical Sciences, Nagasaki 852-8501, Japan; seiko-n@nagasaki-u.ac.jp (S.N.); tmaeda@nagasaki-u.ac.jp (T.M.); 2 Department of Cardiovascular Disease Prevention, Osaka Center for Cancer and Cardiovascular Diseases Prevention, Osaka 537-8511, Japan; 3 Department of Community Medicine, Nagasaki University Graduate School of Biomedical Sciences, Nagasaki 852-8523, Japan; shin-ya@nagasaki-u.ac.jp (S.-Y.K.); y-noguti@nagasaki-u.ac.jp (Y.N.); ynagata1961@nagasaki-u.ac.jp (Y.N.); 4 Leading Medical Research Core Unit, Nagasaki University Graduate School of Biomedical Sciences, Nagasaki 852-8523, Japan; naomin@nagasaki-u.ac.jp; 5 Nagasaki University Health Center, Nagasaki 852-8521, Japan; 6 Division of Promotion of Collaborative Research on Radiation and Environmental Health Effects, Atomic Bomb Disease Institute, Nagasaki University, Nagasaki 852-8523, Japan

**Keywords:** subclinical hypothyroidism, thyroid stimulating hormone, triiodothyronine, HbA1c, renal function, GFR

## Abstract

Subclinical hypothyroidism (SCH) was reported to be associated with accelerating endothelial dysfunction, which is recognized as one of the upstream mechanisms that leads to glomerular injury (lower glomerular filtration rate (GFR)). SCH was also reported to be associated with hyperglycemia, which is associated with higher hemoglobin A1c (HbA1c) levels and induces endothelial dysfunction. Therefore, SCH status could influence the association between HbA1c and reduced eGFR. To clarify those associations, we conducted a prospective study of 1580 Japanese individuals who participated in an annual health check-up in 2014 with 2.8 years of follow-up. All participants had free triiodothyronine (T3) and free thyroxine (T4) levels in the normal range. Among study participants, 88 were diagnosed as having SCH. Even though no significant correlation was observed between HbA1c and annual change in estimated GFR among participants without SCH (multi-adjusted standardized parameter estimate (β) = 0.03, *p* = 0.250), a significant inverse association was observed among participants with SCH (β = −0.26, *p* = 0.014). When those analyses were performed among participants who were not taking glucose lowering medication, the observed associations were essentially the same: β = 0.03, *p* = 0.266 for participants without SCH and β = −0.32, *p* = 0.006 for participants with SCH, respectively. Therefore, SCH status could influence the association between HbA1c and renal function.

## 1. Introduction

Subclinical hypothyroidism (SCH), which is defined as elevated levels of thyroid-stimulating hormone (TSH) with free triiodothyronine (T3) and thyroxine (T4) levels in the normal range, was reported to be associated with accelerating endothelial dysfunction [1]. Recent studies have reported a close connection between thyroid hormones and endothelial maintenance. Thyroid hormones directly stimulate hematopoietic stem cells [2], which can differentiate into endothelial progenitor cells [3,4] that can promote thyroid follicle formation [5,6]. Since endothelial progenitor cells play an important role in endothelial repair [3,4,7] and thyroid follicle cells produce thyroid hormones, the production of thyroid hormones might be associated with endothelial repair capacity. SCH could be associated with accelerating endothelial dysfunction [1].

On the other hand, hyperglycemia, which is associated with higher hemoglobin A1c (HbA1c) levels, is known to induce endothelial dysfunction [8]. Endothelial dysfunction is recognized as one of the upstream mechanisms that leads to glomerular injury, which is associated with reducing the glomerular filtration rate (GFR) [9]. Therefore, higher HbA1c levels could be associated with reduced renal function [10]. Appropriate endothelial repair might mask this association between HbA1c levels and reduced renal function while the presence of SCH might indicate inappropriate endothelial repair.

Therefore, we hypothesized that higher HbA1c levels are significantly associated with reduced renal function as evaluated by annual changes in estimated GFR (eGFR) in individuals with SCH but not in individuals without SCH. To clarify these associations, we conducted a prospective study with a mean follow-up of 2.8 years (standard deviation (SD), 0.5; range 0.8–3.2) of 1580 Japanese individuals with in normal thyroid hormone (i.e., free T3 and free T4 levels in the normal range) aged 40–74 years who participated in an annual health check-up in 2014.

## 2. Materials and Methods

### 2.1. Study Population

Methods related to the present risk survey, including evaluation of thyroid function, have been described elsewhere [11]. We ensured that participants understood the study’s objectives and provided informed consent. This study was approved by the ethics committee of the Nagasaki University Graduate School of Biomedical Sciences (project registration number: 14051404).

The study population comprised 1883 Japanese individuals between the ages of 40 and 74 years from Saza town in western Japan who underwent an annual medical check-up in 2014, as recommended by the Japanese government.

To avoid the influence of thyroid disease, we excluded all participants with a history of thyroid disease (*n* = 60). We also excluded participants with missing data on thyroid function, such as TSH, free T3, and free T4 (*n* = 17), and participants with abnormal free T3 and free T4 levels (*n* = 77). In addition, participants without baseline HbA1c or eGFR data (*n* = 2) and participants without eGFR data in 2015–2017 were also excluded (*n* = 147). The remaining 1580 participants, with a mean age of 60.9 years (SD, 8.9; range 40–74), were included in the study.

### 2.2. Data Collection and Laboratory Measurments

Trained interviewers obtained information on the use of glucose-lowering medication. A fasting blood sample was collected. TSH, free T3, and free T4 levels were measured using a chemiluminescent immunoassay at the LSI Medience Corporation (Tokyo, Japan). The normal range for free T3 (2.1–4.1 pg/mL), free T4 (1.0–1.7 ng/dL), and TSH (0.39–4.01 μIU/mL) using this method was demonstrated elsewhere [12]. SCH was defined as TSH >4.01 μIU/mL and normal free T3 and free T4 levels. By following the report from the committee of the Japan Diabetes Society, we also defined diabetes as HbA1c ≥6.5% and/or taking glucose lowering medication [13].

Serum creatinine concentrations were measured using a standard laboratory procedure at SRL, Inc. (Tokyo, Japan). eGFR was calculated with an established method recently adapted by a working group of the Japanese Chronic Kidney Disease Initiative [14]: eGFR (mL/min/1.73 m^2^) = 194 × (serum creatinine (enzyme method))^−1.094^ × (age)^−0.287^ × (0.739 for women). Annual change in eGFR (annual ΔeGFR) was calculated as annual ΔeGFR ([mL/min/1.73 m^2^]/year) = [endpoint eGFR (mL/min/1.73 m^2^)–baseline eGFR (mL/min/1.73 m^2^)]/[endpoint date–baseline date].

### 2.3. Statistical Analysis

Characteristics of the study participants by SCH status were expressed as means ±SD, except for gender and glucose lowering medication use. Those variables were expressed as proportions. 

Differences between mean values or proportions of characteristics were analyzed in relation to SCH status. Significant differences were evaluated using the *t*-test for continuous variables and the χ^2^ test for categorical variables.

Simple correlation analysis and multivariable linear regression analysis of annual decrease of eGFR based on diabetes status and SCH status adjusting for relevant confounding factors were performed. Renal function at baseline might influence annual ΔeGFR. Therefore, for the multivariable linear regression analysis, adjustments were made for sex, age, free T_3_ (pg/mL), TSH (μIU/mL), and eGFR (mL/min/1.73 m^2^).

Since diabetes could influence the main results, analysis limited to participants without diabetes was performed.

To avoid the influence of age on the present results, by using the age-matched sample, the association between HbA1c and annual ΔeGFR among participants without SCH was evaluated.

Glucose-lowering medication use could act as a strong confounder in the main analysis. As a sensitivity analysis, we also performed these analyses in a subset of participants who were not taking glucose-lowering medication.

All statistical analysis was performed with SAS for Windows, version 9.4 (SAS Inc., Cary, NC, USA). Values of *p* < 0.05 were regarded as statistically significant.

## 3. Results

### 3.1. Characteristics of Study Population by Subclinical Hypothyroidism (SCH) Status

Among the study participants, 88 were diagnosed as having SCH and 140 were diagnosed as having diabetes. The characteristics of study participants by SCH status are shown in Table 1.

Compared with participants without SCH, participants with SCH had significantly higher values of TSH and HbA1C. Participants with SCH had a significantly higher prevalence of diabetes, glucose-lowering medication use and significantly lower values of free T4 and eGFR. Participants with SCH had lower free T3 values than participants without SCH, but this difference was not statistically significant.

**Figure 1:**
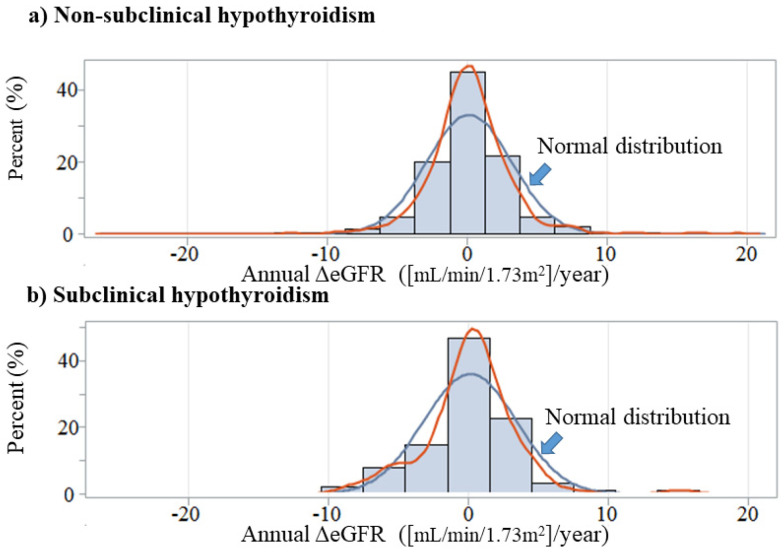
Annual change in eGFR by subclinical hypothyroidism status. Annual ΔeGFR: annual change in estimated glomerular filtration rate ([mL/min/1.73m^2^]/year).

Figure 1 shows the distribution of annual ΔeGFR by SCH status. Participants with and without SCH had essentially the same associations.

### 3.2. Correlations between Annual Change in Estimated Glomerular Filtration Rate (ΔeGFR) and Hemoglobin A1c (HbA1c) in Relation to Diabetes

Correlations between annual ΔeGFR and HbA1c for total participants and stratified by diabetes are shown in Table 2. Among total participants, both by simple correlation analysis and by multiple linear regression analysis, no significant associations between HbA1c and annual ΔeGFR were observed. These associations were also observed when the analyses were limited to participants with diabetes and limited to those without diabetes (Table 2).

### 3.3. Correlations between Annual ΔeGFR and HbA1c by SCH Status among Participants without Diabetes

Correlations between annual ΔeGFR and HbA1c by SCH status among participants without diabetes are shown in Table 2. In the simple correlation analysis, there was no significant correlation between HbA1c and annual ΔeGFR in participants with and without SCH. After adjusting for sex, age, free T3, TSH, and eGFR in a multivariable linear regression analysis, a significant inverse association between HbA1c and annual ΔeGFR was observed in participants with SCH but not in participants without SCH (Table 3).

### 3.4. Correlations between Annual ΔeGFR and HbA1c by SCH Status

**Figure 2:**
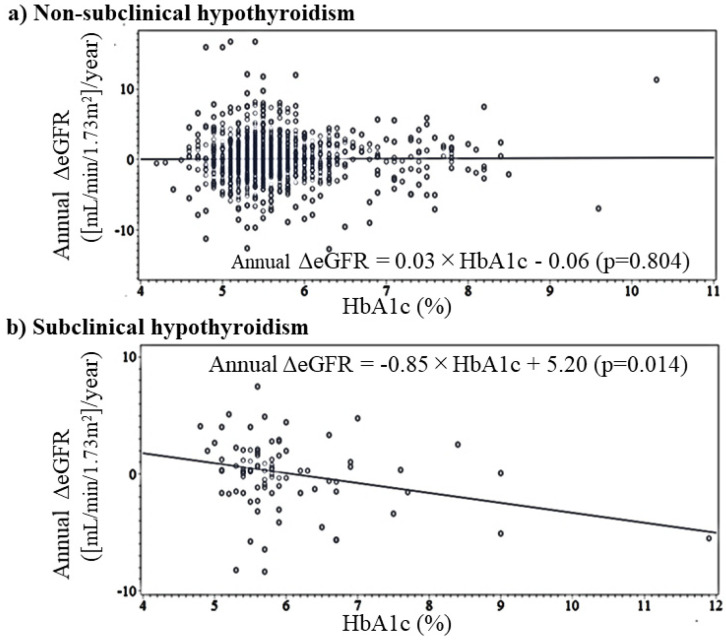
Simple linear regression analysis of HbA1c and annual change in eGFR by subclinical hypothyroidism status. Annual ΔeGFR: annual change in estimated glomerular filtration rate ([mL/min/1.73m^2^]/year).

Correlations between annual ΔeGFR and HbA1c by SCH status are shown in Table 2. In the simple correlation analysis, there was no significant correlation between HbA1c and annual ΔeGFR in participants without SCH and a significant inverse association in participants with SCH. This inverse association shows linear association (Figure 2). After adjusting for sex, age, TSH, and free T3 in a multivariable linear regression analysis, the associations were essentially the same (Table 4).

### 3.5. Correlations between Annual ΔeGFR and HbA1c among Non-SCH Status by Using Age-Matched Model

Table 5 shows the correlations between annual ΔeGFR and HbA1c among non-SCH by using an age-matched model. In the simple correlation analysis, there was no significant correlation between HbA1c and annual ΔeGFR. This association was unchanged even when further adjusted for known confounding factors.

### 3.6. Correlations between ΔeGFR and HbA1c by SCH Status among Participants Who Were Not Taking Glucose-Lowerung Medication

Since glucose-lowering medication use could influence HbA1c values, it might act as a strong confounding factor in the present analysis. Thus, we performed an additional analysis of participants who were not taking glucose-lowering medication. The associations were essentially the same (Table 6).

## 4. Discussion

**Figure 3:**
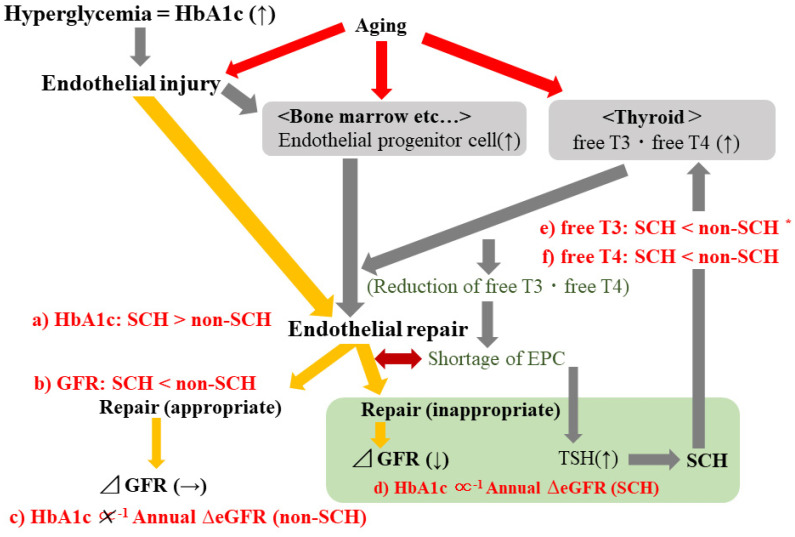
Potential mechanisms underlying the present results. Associations shown in red (**a**–**f**) were observed in the present study. TSH, thyroid-stimulating hormone; T3, triiodothyronine; T4, thyroxine; eGFR, estimated glomerular filtration rate; SCH, subclinical hypothyroidism; Annual ΔeGFR: annual change in estimated glomerular filtration rate ([mL/min/1.73m^2^]/year). * Non-statistically significant association.

The main finding of the present longitudinal study is that higher HbA1c is significantly associated with reduced eGFR in participants with SCH but not in those without SCH. Even when limited to participants who were not taking glucose-lowering medication, the associations were essentially the same. However, the mechanisms underlying the present results have not yet been clarified. We performed a multi-faceted analysis to clarify the mechanisms that could potentially explain the present results. A summary of the potential mechanism is shown in Figure 3. Associations shown in red (Figure 3a–f) were observed in the present study. Endothelial repair activated by endothelial injury, which is associated with hyperglycemia, might play an important role.

Previous case-control studies that involved patients with SCH (diagnosed as mild elevation in TSH levels with normal free T3 and free T4 levels) and age- and gender-matched normal controls (defined as TSH, free T3, and free T4 levels being in the normal range) showed a significant positive correlation between insulin resistance as evaluated by homeostasis model assessment-insulin resistance (HOMA-IR) and TSH [15]. Increased insulin resistance is a well-known cause of type 2 diabetes. In the present study, participants with SCH had significantly higher baseline values of HbA1c than participants without SCH (Table 1). Furthermore, the analysis that was limited to participants who were not taking glucose-lowering medication showed essentially the same association; the corresponding HbA1c values were 5.8 ± 0.9% for participants with SCH and 5.5 ± 0.5% for participants without SCH (*p* < 0.001). Therefore, SCH could be associated with higher baseline HbA1c (Table 1, Figure 3a).

A previous meta-analysis reported a positive association between SCH and a higher risk of chronic kidney disease (CKD) [16]. Furthermore, a previous hospital-based case-control study with 3270 euthyroid patients with type 2 diabetes and 545 patients with type 2 diabetes and SCH reported that SCH could be a significant risk factor for CKD in patients with diabetes [17]. The findings of these studies are compatible with our present results that showed baseline renal function as evaluated by baseline eGFR in participants with SCH was significantly lower than that of participants without SCH (Table 1, Figure 3b). Our longitudinal analysis revealed a significant correlation between baseline HbA1c and renal function as evaluated by annual ΔeGFR only among participants with SCH (Table 3, Table 4 and Table 6, Figure 3c,d). However, a high prevalence of diabetes among SCH [18] could not explain the significant association between HbA1c and annual ΔeGFR in participants with SCH. By using multivariable model, a significant association between HbA1c and annual ΔeGFR was observed even when the analysis was performed limited to SCH without diabetes (Table 3). Furthermore, the status of diabetes did not influence the association between HbA1c and annual ΔeGFR (Table 2). Therefore, the status of diabetes might not act as a determinant on the association between HbA1c and annual ΔeGFR.

Hyperglycemia, which is associated with higher HbA1c levels, is known to induce endothelial dysfunction [8]. Endothelial dysfunction is recognized as one of the upstream mechanisms that leads to glomerular injury, which is associated with lower eGFR [9]. Since endothelial progenitor cells contribute to endothelial repair, the presence of endothelial injury stimulates the production of endothelial progenitor cells [7]. Our previous studies showed that higher levels of circulating endothelial progenitor cells (CD34-positive cells) are associated with higher HbA1c values [7,19,20]. However, the number of endothelial progenitor cells and their functions are reported to be decreased with advancing CKD [21]. Reduction in the number of endothelial cells due to consumption [22] might lead to an inverse relationship between endothelial progenitor cell count and CKD.

On the other hand, thyroid hormones directly stimulate hematopoietic stem cells, which differentiate into endothelial progenitor cells [2]. Therefore, a relative shortage of endothelial progenitor cells could be associated with increased TSH production, which results in SCH. In other words, participants with SCH might have a relative deficiency of endothelial progenitor cells that results in a lower ability to maintain renal function.

In addition, lower levels of thyroid hormones due to consumption could occur in participants with aggressive endothelial repair since thyroid hormones directly stimulate hematopoietic stem cells [2]. Thyroid hormone levels are lower in participants with SCH than in participants without SCH, as we showed in the present study (Table 1, Figure 3e,f). Hematopoietic activity in the bone marrow declines with age [23] and aging is also a well-known cause of endothelial injury [24,25]. However, decreased thyroid function, as well as TSH levels may contribute to the increased lifespan [26]. Demands for thyroid hormone might decrease with aging [27]. TSH levels could increase with the aging process, regardless of whether there is actual thyroid disease [28]. However, the influence of age on the present main associations should be limited. In the present study, participants with SCH were older than participants without SCH, even though the difference was not statistically significant (Table 1). Furthermore, in the main results, significant association between HbA1c and annual ΔeGFR were observed among participants with SCH even after adjusted for age (Table 3, Table 4 and Table 6).

Therefore, inappropriate endothelial repair activity might lead to the development of SCH. To clarify this mechanism, further investigation with information about endothelial progenitor cells is necessary.

From the clinical perspective, the present study demonstrated that SCH status could act as an effect modifier on the association between HbA1c and renal function. Therefore, treatment of SCH could be an efficient strategy for preventing diabetic nephropathy. Furthermore, even though further investigation is necessary, this study also suggests that the presence of SCH could indicate the presence of inappropriate endothelial repair.

Potential limitations of this study warrant consideration. Excessive consumption of iodine and the presence of autoimmune antibodies or congenital factors are known to be associated with thyroid function. Those factors could act as confounders but we do not have any data on them. However, our present analysis was performed among participants without any history of thyroid disease. Even though endothelial progenitor cells might play an important role in the mechanisms underlying the present results, we have no data about endothelial progenitor cells because of the difficulty in measuring those cells in routine health examinations. Further studies with those data are necessary.

## 5. Conclusions

In conclusion, the disadvantage of elevated HbA1c on renal function is observed only among participants with SCH. SCH status could act as an effect modifier on the association between HbA1c and renal function.

## Figures and Tables

**Table 1:** Characteristics of study participants.

	Subclinical Hypothyroidism		*p*
	(−)	(+)	
No of participants	1492	88	
Men, %	36.7	39.8	0.566
Age, year	60.8 ± 8.9	62.6 ± 8.9	0.074
free T3, (2.1–4.1) pg/mL	3.2 ± 0.3	3.1 ± 0.3	0.084
free T4, (1.0–1.7) ng/dL	1.3 ± 0.2	1.2 ± 0.2	<0.001
TSH, (0.39–4.01) μIU/mL	1.7 ± 0.8	5.7 ± 1.7	<0.001
Diabetes, %	8.2	20.5	<0.001
Glucose lowering medication use, %	5.4	12.5	<0.001
HbA1c, %	5.6 ± 0.6	6.0 ± 1.0	<0.001
Serum creatinine, mg/dL	0.75 ± 0.33	0.82 ± 0.32	0.069
eGFR, mL/min/1.76m^2^	71.4 ± 12.9	67.0 ± 15.0	0.002

Values are means ± SD. Normal ranges are given in parentheses. TSH, thyroid-stimulating hormone; T3, triiodothyronine; T4, thyroxine; HebA1, hemoglobin A1c; eGFR, estimated glomerular filtration rate.

**Table 2:** Simple correlation analysis and multivariable linear regression analysis of annual ΔeGFR and relevant factors for total and by diabetes status.

	Simple Correlation Analysis	Multiple Linear Regression Analysis		
	r (*p*)	Β	β	*p*
**Total**				
No of participants	1580			
Sex (Men)	−0.07 (*p* = 0.008)	−0.51	−0.08	0.020
Age	−0.03 (*p* = 0.320)	−0.02	−0.06	0.028
free T3	−0.01 (*p* = 0.646)	0.30	0.03	0.240
TSH	−0.002 (*p* = 0.943)	−0.05	−0.02	0.396
eGFR	0.13 (*p <* 0.001)	−0.04	−0.16	<0.001
HbA1c	−0.02 (*p* = 0.417)	−0.01	−0.002	0.938
**Non-diabetes**				
No of participants	1440			
Sex (Men)	−0.07 (*p* = 0.013)	−0.45	−0.07	0.080
Age	−0.03 (*p* = 0.196)	−0.02	−0.07	0.012
free T3	−0.01 (*p* = 0.695)	0.33	0.04	0.202
TSH	0.02 (*p* = 0.528)	0.002	0.001	0.979
eGFR	−0.13 (*p* < 0.001)	−0.04	−0.16	<0.001
HbA1c	0.001 (*p* = 0.970)	−0.01	−0.001	0.982
**Diabetes**				
No of participants	140			
Sex (Men)	−0.11 (*p* = 0.208)	−1.11	−0.13	0.123
Age	0.09 (*p* = 0.284)	0.02	0.03	0.710
free T3	−0.03 (*p* = 0.721)	0.18	0.01	0.870
TSH	−0.08 (*p* = 0.358)	−0.33	−0.14	0.113
eGFR	−0.17 (*p* = 0.048)	−0.06	−0.21	0.021
HbA1c	0.01 (*p* = 0.884)	0.26	0.06	0.530

ΔGFR, change in estimated glomerular filtration rate; eGFR, estimated glomerular filtration rate; T3, triiodothyronine; TSH, thyroid-stimulating hormone; HebA1, hemoglobin A1c; r (*p*), simple correlation coefficient (*p* value); Β, parameter estimate; β, standardized parameter estimate.

**Table 3:** Simple correlation analysis and multivariable linear regression analysis of annual ΔeGFR and relevant factors by subclinical hypothyroidism among participants without diabetes.

	Simple Correlation Analysis	Multiple Linear Regression Analysis		
	r (*p*)	Β	β	*p*
**Non-Subclinical Hypothyroidism**				
No of participants	1370			
Sex (Men)	−0.08 (*p* = 0.004)	−0.51	−0.09	0.003
Age	−0.05 (*p* = 0.094)	−0.03	−0.09	0.003
free T3	−0.01 (*p* = 0.718)	0.38	0.04	0.152
TSH	0.02 (*p* = 0.574)	−0.02	−0.01	0.857
eGFR	−0.14 (*p* < 0.001)	−0.04	−0.17	<0.001
HbA1c	0.01 (*p* = 0.759)	0.08	0.01	0.736
**Subclinical Hypothyroidism**				
No of participants	70			
Sex (Men)	0.13 (*p* = 0.266)	1.72	0.26	0.055
Age	0.14 (*p* = 0.239)	0.10	0.27	0.038
free T3	0.001 (*p* = 0.996)	−1.24	−0.11	0.386
TSH	−0.12 (*p* = 0.338)	−0.29	−0.14	0.248
eGFR	0.07 (*p* = 0.557)	0.03	0.11	0.416
HbA1c	−0.17 (*p* = 0.167)	−2.58	−0.25	0.049

ΔGFR, change in estimated glomerular filtration rate; eGFR, estimated glomerular filtration rate; T3, triiodothyronine; TSH, thyroid-stimulating hormone; HebA1, hemoglobin A1c; r (*p*), simple correlation coefficient (*p* value); Β, parameter estimate; β, standardized parameter estimate.

**Table 4:** Simple correlation analysis and multivariable linear regression analysis of annual ΔeGFR and relevant factors by subclinical hypothyroidism status.

	Simple Correlation Analysis	Multiple Linear Regression Analysis		
	r (*p*)	Β	β	*p*
**Non-Subclinical Hypothyroidism**				
No of participants	1492			
Sex (Men)	−0.08 (*p* = 0.002)	−0.57	−0.09	<0.001
Age	−0.04 (*p* = 0.161)	−0.03	−0.08	0.003
free T3	−0.01 (*p* = 0.592)	0.34	0.04	0.194
TSH	0.01 (*p* = 0.670)	−0.05	−0.01	0.641
eGFR	−0.15 (*p* < 0.001)	−0.04	−0.18	<0.001
HbA1c	0.01 (*p* = 0.804)	0.16	0.03	0.250
**Subclinical Hypothyroidism**				
No of participants	88			
Sex (Men)	0.06 (*p* = 0.580)	0.55	0.08	0.472
Age	0.15 (*p* = 0.163)	0.07	0.19	0.095
free T3	0.03 (*p* = 0.809)	−0.53	−0.05	0.674
TSH	−0.10 (*p* = 0.360)	−0.10	−0.05	0.625
eGFR	0.09 (*p* = 0.429)	0.04	0.16	0.166
HbA1c	−0.26 (*p* = 0.014)	−0.86	−0.26	0.014

ΔGFR, change in estimated glomerular filtration rate; eGFR, estimated glomerular filtration rate; T3, triiodothyronine; TSH, thyroid-stimulating hormone; HebA1, hemoglobin A1c; r (*p*), simple correlation coefficient (*p* value); Β, parameter estimate; β, standardized parameter estimate.

**Table 5:** Simple correlation analysis and multiple linear regression analysis of annual ΔeGFR and relevant factors among non-subclinical hypothyroidism by using age-matched sample.

	Simple Correlation Analysis	Multiple Linear Regression Analysis		
	r (*p*)	Β	β	*p*
**Non-Subclinical Hypothyroidism**				
No of participants	176			
Sex (Men)	−0.11 (*p* = 0.138)	−0.83	−0.14	0.107
Age	0.03 (*p* = 0.690)	0.002	0.01	0.937
free T3	−0.09 (*p* = 0.239)	−0.26	−0.03	0.745
TSH	0.004 (*p* = 0.959)	−0.09	−0.02	0.774
eGFR	−0.14 (*p* = 0.080)	−0.03	−0.13	0.112
HbA1c	0.11 (*p* = 0.173)	0.55	0.13	0.105

T3, triiodothyronine; TSH, thyroid-stimulating hormone; eGFR, glomerular filtration rate; HebA1, hemoglobin A1c; r(*p*), simple correlation coefficient (*p* value); Β, parameter estimate; β, standardized parameter estimate.

**Table 6:** Simple correlation analysis and multivariable linear regression analysis of annual ΔeGFR and relevant factors by subclinical hypothyroidism status among participants who were not taking glucose-lowering medication.

	Simple Correlation Analysis	Multiple Linear Regression Analysis		
	r (*p*)	Β	β	*p*
**Non-Subclinical Hypothyroidism**				
No of participants	1411			
Sex (Men)	−0.08 (*p* = 0.002)	−0.54	−0.09	0.002
Age	−0.04 (*p* = 0.115)	−0.03	−0.08	0.003
free T3	−0.02 (*p* = 0.566)	0.30	0.03	0.267
TSH	0.01 (*p* = 0.746)	−0.04	−0.11	0.690
eGFR	−0.13 (*p* < 0.001)	−0.04	−0.16	<0.001
HbA1c	0.02 (*p* = 0.424)	0.19	0.03	0.266
**Subclinical Hypothyroidism**				
No of participants	77			
Sex (Men)	0.17 (*p* = 0.139)	1.63	0.24	0.049
Age	0.19 (*p* = 0.101)	0.09	0.24	0.050
free T3	0.04 (*p* = 0.760)	−1.52	−0.14	0.261
TSH	−0.07 (*p* = 0.550)	−0.33	−0.16	0.172
eGFR	−0.001 (*p* = 0.993)	0.03	0.12	0.331
HbA1c	−0.28 (*p* = 0.015)	−1.14	−0.32	0.006

ΔGFR, change in estimated glomerular filtration rate; eGFR, estimated glomerular filtration rate; T3, triiodothyronine; TSH, thyroid-stimulating hormone; HbA1c, hemoglobin A1c; r (*p*), simple correlation coefficient (*p* value); Β, parameter estimate; β, standardized parameter estimate.

## Data Availability

We cannot publicly provide individual data due to participant privacy, according to ethical guidelines in Japan. Additionally, the informed consent obtained does not include a provision for publicity-sharing data. Qualifying researchers may apply to access a minimal dataset by contacting Prof Naomi Hayashida, Principal Investigator, Division of Promotion of Collaborative Research on Radiation and Environment Health Effects, Atomic Bomb Disease Institute, Nagasaki University, Nagasaki, Japan at naomin@nagasaki-u.ac.jp. Or, please contact the office of data management at ritouken@vc.fctv-net.jp. Information for where data request is also available online: https://www.genken.nagasaki-u.ac.jp/dscr/message/ and http://www.med.nagasaki-u.ac.jp/cm/ (accessed on 12 January 2022).
